# A Mixed Methods Evaluation of Early Childhood Abuse Prevention Within Evidence-Based Home Visiting Programs

**DOI:** 10.1007/s10995-018-2530-1

**Published:** 2018-05-31

**Authors:** M. Matone, K. Kellom, H. Griffis, W. Quarshie, J. Faerber, P. Gierlach, J. Whittaker, D. M. Rubin, P. F. Cronholm

**Affiliations:** 1 PolicyLab, The Children’s Hospital of Philadelphia, Philadelphia, PA USA; 2 Division of General Pediatrics, The Children’s Hospital of Philadelphia, Philadelphia, PA USA; 3 Perelman School of Medicine, University of Pennsylvania, Philadelphia, PA USA; 4 Mixed Methods Research Lab, University of Pennsylvania, Philadelphia, PA USA; 5 Department of Family Medicine and Community Health, University of Pennsylvania, Philadelphia, PA USA; 6 Center for Public Health Initiatives, University of Pennsylvania, Philadelphia, PA USA; 7 Leonard Davis Institute of Health Economics, University of Pennsylvania, Philadelphia, PA USA; 8 Roberts Center for Pediatric Research, 2716 South Street, Philadelphia, PA 19146 USA

**Keywords:** Home visiting, Maternal and child health, Child maltreatment, Mixed methods research

## Abstract

*Objectives* In this large scale, mixed methods evaluation, we determined the impact and context of early childhood home visiting on rates of child abuse-related injury. *Methods* Entropy-balanced and propensity score matched retrospective cohort analysis comparing children of Pennsylvania Nurse–Family Partnership (NFP), Parents As Teachers (PAT), and Early Head Start (EHS) enrollees and children of Pennsylvania Medicaid eligible women from 2008 to 2014. Abuse-related injury episodes were identified in medical assistance claims with ICD-9 codes. Weighted frequencies and logistic regression odds of injury within 24 months are presented. In-depth interviews with staff and clients (n = 150) from 11 programs were analyzed using a modified grounded theory approach. *Results* The odds of a healthcare encounter for early childhood abuse among clients were significantly greater than comparison children (NFP: 1.32, 95% CI [1.08, 1.62]; PAT: 4.11, 95% CI [1.60, 10.55]; EHS: 3.15, 95% CI [1.41, 7.06]). Qualitative data illustrated the circumstances of and program response to client issues related to child maltreatment, highlighting the role of non-client caregivers. All stakeholders described curricular content aimed at prevention (e.g. positive parenting) with little time dedicated to addressing current or past abuse. Clients who reported a lack of abuse-related content supposed their home visitor’s assumption of an absence of risk in their home, but were supportive of the introduction of abuse-related content. Approach, acceptance, and available resources were mediators of successfully addressing abuse. *Conclusions for Practice* Home visiting aims to prevent child abuse among high-risk families. Adequate home visitor capacity to proactively assess abuse risk, deliver effective preventive curriculum with fidelity to caregivers, and access appropriate resources is necessary.

**Electronic supplementary material:**

The online version of this article (10.1007/s10995-018-2530-1) contains supplementary material, which is available to authorized users.

## Significance Statement

*What is already known on this topic?* Reducing child maltreatment is a public health priority. At present, home visiting represents the primary available prevention strategy for child maltreatment; however, evaluations of home visiting success on this outcome have been varied.

*What does this study add?* Our mixed methods evaluation pairs a large administrative dataset that allows us to measure child abuse outcomes reliably across multiple home visiting programs with qualitative interviews with key stakeholders to demonstrate and explore home visiting’s impact on child maltreatment. We find no evidence of positive program effects but identify explanatory implementation factors potentially limiting program effectiveness.

## Introduction

Child maltreatment is a serious public health problem (Children’s Bureau 2016) resulting in innumerable short and long-term health consequences including trauma, adverse physical and mental/behavioral health, changes to brain architecture and development, challenges to educational achievement, and reduced social-emotional functioning and relational attachment (Deutsch et al. 2015; Merrick and Latzman 2014). Unfortunately, prevention is challenging to achieve in public health programming, in part due to the level of support needed to overcome risk factors and bolster protective factors for families and communities (Agran et al. 2003; Frioux et al. 2014; Wood et al. 2012). Early childhood maltreatment prevention programs (i.e. home visitation) intervene at a critical period when children are most at risk (Schatz and Lounds 2007) and promote prevention though strengthening protective factors within a family and connecting families with community services. Programs teach approaches to child rearing, decreasing parental stress, provide guidance on reducing childhood hazard exposures, and serve a monitoring function for identifying and responding to maltreatment (Gomby et al. 1999).

Inconclusive and at times conflicting clinical trials and post-implementation studies regarding home visiting’s impact on child maltreatment rates have highlighted the need for further attention and evaluation (Institute of Medicine and National Research Council 2014; Rubin et al. 2014). The varied success in reducing child maltreatment necessitates further investigation into the conditions under which home visiting programs can achieve prevention, and for whom (Howard and Brooks-Gunn 2009).

Early results from home visiting evaluations suggested efficacy in decreasing child maltreatment. Notably, a randomized controlled trial of the Nurse–Family Partnership (NFP) resulted in 80% fewer injury and ingestion-related doctor visits in intervention group (though non-significant at 2 years) (Olds et al. 1994). However, following wide-scale replication of evidence-based home visiting programs, data are limited in supporting the effectiveness in preventing abuse (Matone et al. 2012). A 2013 review of home visitation programs found two programs reduced long-term child maltreatment reports or death contrasted by four other randomized trials showing no associated effect of home visitation on child maltreatment reports (Nelson et al. 2013; Rubin et al. 2014).

As home visiting programs have scaled in the context of the federal Maternal, Infant, and Early Childhood Home Visiting program (MIECHV) (“Patient Protection and Affordable Care Act” 2010), it is important to refine our understanding of their role and mechanism, and opportunities for maltreatment prevention. In this large scale, mixed methods evaluation, we aimed to estimate the effectiveness of three MIECHV-funded home visiting models in Pennsylvania (PA) on early childhood maltreatment ascertained from clinical diagnoses in emergency department and inpatient healthcare encounters. A concurrent qualitative analysis of interviews with program staff and clients explores the content of and response to curriculum related to child maltreatment.

## Data and Methods

This study was performed within the Commonwealth of Pennsylvania (PA) MIECHV evaluation. The study followed a partially mixed, concurrent, equal status design, in which qualitative and quantitative data were analyzed separately and mixed at the stage of interpretation (Leech and Onwuegbuzie 2009). The study was approved by PA’s Department of Human Services with human subjects approval by the Children’s Hospital of Philadelphia’s Institutional Review Board.

### Quantitative Data

#### Analytic Sample

Data was obtained for clients enrolled in MIECHV funded PA nurse–family partnership (NFP, n = 22), parents as teachers (PAT, n = 9), or early head start (EHS, n = 7) from 2008 to 2014. Clients were matched to local-area non-client women (comparisons) who (1) had similarly aged children identified in birth certificate files and (2) resided in the same local implementing agency catchment area (i.e., county or multi-county service area). Inclusion criteria for clients and comparisons were as follows: (1) child affiliated with MIECHV program enrollment was identifiable in PA birth certificate files and (2) child affiliated with MIECHV program enrollment had enrollment in the state medical assistance program (Medicaid) during the outcome observation period.

Clients and potential comparisons were identified in a multisource administrative data file linked using an iterative deterministic approach reliant on unique identifiers constructed from social security numbers, names, and dates of birth that included program enrollment, vital statistics (birth and death), welfare eligibility, and medical assistance claim files (Dusetzina et al. 2014).

#### Quasi-experimental Design

The primary analysis examined if the prevalence and rate of child abuse episodes significantly differed between program clients and comparison women for NFP, EHS, and PAT programs separately. Two primary quasi-experimental methods were used for causal inference related to program effect on child abuse: entropy balancing for NFP and propensity score matching (PSM) for EHS and PAT. Both analytic approaches are widely used for obtaining covariate balance in observational data, but neither approach was suitable for all three program model analyses for the following reasons.

First, PSM has a disadvantage of dropping subjects unable to be matched to counterparts, which creates a biased sample. In the case of this study, PSM did not retain a generalizable subset of the clients in the NFP analysis (specifically, young mothers in rural areas were disproportionately dropped in attempted PSM). Entropy balancing retained all cases and was the approach used for NFP. While PSM was not the optimal approach for NFP, it was chosen for the EHS and PAT analyses because it allowed for standardized follow-up time in the outcome observation windows of matched sets of clients and comparisons. This is a critical analytic design feature for programs without standardized enrollment at a point in time. Unlike NFP, which uniformly enrolls clients into the program prior to a child’s birth, EHS and PAT programs do not uniformly enroll at a particular age. Therefore, for each client, PSM allowed for the identification of comparisons with similarly aged children at the time of program enrollment. The analysis then standardized observation periods for outcome ascertainment within matched sets of clients and comparisons using the client’s child’s age of enrollment and length of time in the program as the reference point (e.g., if client enrolled child at 3 months and was observed through month 27, all comparison children for that client are observed for months 3 through 27). This level of modeling flexibility is not possible with entropy balancing, but was also not necessary for NFP given the requirement of prenatal enrollment, which serves as a standardization (i.e., all client and comparison children begin observation at birth).

Both entropy balancing and PSM were performed within local implementing agency catchment areas (Matone et al. 2012) to address the possibility that there is confounding by geography (i.e., the outcomes might vary across sites at a community level beyond maternal-level characteristics). Catchments included each implementing agency’s county and contiguous counties. Clients enrolled in a program in a particular catchment were matched to comparison women living in the same catchment. Entropy balancing and PSM were performed within catchments, and then the samples were aggregated.

#### Description of PSM for EHS and PAT

PSM is a matching technique for observational data that mimics a randomized control trial by creating pairs (or sets) from clients and comparison women with similar values of the propensity score (Stuart 2010). Multivariable logistic regression models estimate the probability of program participation using available maternal sociodemographic and clinical characteristics—from birth certificate: mother’s age at birth (continuous), race/ethnicity (white/black/Hispanic/other), maternal education (< high school/high school or greater), gestational age (continuous), smoking prior to pregnancy (y/n); from welfare eligibility files: receipt of Temporary Assistance for Needy Families (TANF) or supplemental nutrition program prior to or during the first trimester of pregnancy (y/n); from medical assistance claims: Medicaid eligibility (y/n), maternal diagnosis of substance abuse, depression and/or bipolar disorder in the immediate preconception period or first trimester of pregnancy (y/n). A separate logistic regression model was performed within each of the catchment areas for each local implementing agency. Our matching approach used both caliper and exact matching on covariates to produce matched sets. Any nearest neighbor within a caliper of 0.05 was considered a match (up to a maximum of four comparison women per client). Matching was conducted exactly on catchment area, infant year of birth, and maternal age (< 18 years of age at birth or 18 and older). A threshold of 2.5 absolute percentage points was used to determine balance within each catchment area model. Interaction terms were added to the propensity score model when needed to achieve balance. Analytic weights were developed within matched sets; each comparison woman was given a weight equal to the inverse of the number of comparison women matched to that client and each client was given a weight of 1. These weights were applied to outcome modeling.

#### Description of Entropy Balancing for NFP

Entropy matching is a multivariable weighting technique that creates a balanced sample by reweighting the control group (in this case the comparison women) to have the same covariate distribution as the treatment group (i.e., the clients) using the above described maternal sociodemographic and clinical characteristics. In this approach, specifications for each covariate can be applied as to whether exact balance between the two groups should be achieved on the first moment (mean), second moment (standard deviation), or higher moments (Hainmueller and Xu 2013). As is intended with this methodology, there is automatic balance created between the samples after conducting entropy balancing, so no additional balance checks or model adjustment to create balance was necessary. Covariates used in entropy balancing were the same as included in PSM.

#### Abuse and Injury Episode Creation

The primary outcome for this study was the presence of an abuse episode or high risk injury episode (composite measure) with a secondary outcome that identified the presence of any injury episode. Outcome measures were derived from child Medicaid claims. Episodes were created to conservatively count unique instances of abuse and injury recognizing that multiple claims/encounters may exist for a single event. The methodology of collapsing claim encounters to create episodes is described in Matone et al. 2012 and further in Online Appendix A.

Abuse episodes were those in which an ICD-9 code indicated child abuse (995.50-5, 995.59), as well as high risk injuries (HRI), specific types of severe injuries considered highly suspicious for abuse without the presence of a medical diagnosis of abuse in the medical record. These episodes feature injuries that include fractures of the femur, radius, ulna, tibia, fibula, humerus, ribs, or traumatic brain injuries within the first 24 months of life (without the presence of ICD-9 codes indicating an injury due to a motor vehicle crash) (Wood 2010) (Online Appendix B).

Injury outcomes included: superficial injuries, a composite of dislocation, fracture, and crush injuries, poisonings, and burns identified through ICD-9 codes.

The observation window for episodes were claims during 0 to 24 months of life for NFP cohort and, for EHS and PAT cohorts, 24 months post-enrollment, up to 6 years of life. Right-censoring of episodes occurred for children whose observation periods exceeded the study end period of 2014.

#### Outcome Models

The primary exposure was NFP, PAT, or EHS program participation. For EHS and PAT analyses, a weighted conditional logistic regression model was used to examine the unadjusted association between program participation and the primary outcome. For the NFP analysis, a weighted logistic regression with a random intercept for county was used to estimate the relationship between program participation and the primary outcome, controlling for the variability in the outcome across counties. The presence of abuse or injury prior to enrollment was included as an adjustment covariate in PAT and EHS final outcome models (not applicable for NFP modeling given prenatal enrollment) to account for baseline injury risk.

#### Sensitivity Analyses

Two sensitivity analyses were conducted to test if the relationship found between program participation and abuse outcome was robust to potential confounders that could not be included as covariates in the PSM or entropy balancing model. We tested for confounding between program participation and abuse by maternal psychosocial risk factors by separately including each risk factor in the primary outcome model and examining if the estimated odds ratio effect for the program participation, adjusting for the risk factor, changed by 10% or more. The risk factors ascertained from the literature to be confounders are (1) maternal previous involvement with child protective services (CPS) before pregnancy and (2) intimate partner violence (IPV) measured after conception (Berlin et al. 2011; Eckenrode et al. 2000).

To identify clients involved in CPS, NFP clients and comparisons residing in Philadelphia were linked to county child welfare records via first name, last name, date of birth, and gender. Child welfare systems are administered at the county-level in PA; Philadelphia represents the largest county in the state and produced a sample large enough for sensitivity analysis. For any clients and comparisons successfully linked to child welfare records, dates of protective service were provided. We identified mothers with childhood CPS involvement (prior to pregnancy).

Regarding the second sensitivity analysis, IPV was identified in maternal medical encounters as an ICD-9 code of 995.8x during the observation window of child’s date of conception through the first month of life. Even though we identified IPV in some clients after program enrollment occurred or after the program services could have started, we deemed this time window as most meaningful for measuring IPV for two reasons: (1) to reflect a baseline risk proximal to program enrollment and (2) to increase likelihood of ascertainment in medical assistance files given increased health seeking during pregnancy and increased risk period for IPV. While rates of IPV during pregnancy vary depending on the samples studied and measures used, the prevalence of IPV during pregnancy is elevated compared to women of non-reproductive age and may be increased compared to non-pregnant patients (Hellmuth et al. 2013; Jasinski 2004). Less biased screening (i.e. more universal screening) may occur during the prenatal period due to recommendations by professional organizations, such as the American Congress of Obstetricians and Gynecologists’ (ACOG), that providers should screen all women for IPV at periodic intervals, including during obstetric care (at the first prenatal visit, at least once per trimester, and at the postpartum checkup) (“ACOG Committee Opinion No. 518: Intimate partner violence” 2012).

For each set of primary analyses described above, we ran two additional models—one with a dichotomous covariate for presence of maternal involvement with CPS prior to childbirth and another with a covariate for presence of IPV within child’s date of conception through the first month of life.

#### Presentation of Results

Logistic regression results were expressed as odds ratios (with 95% confidence intervals) and standardized marginal probabilities. All analyses were conducted using SAS version 9.4, Stata version 14.2 and R. Stata’s ebalance package was used for entropy balancing and R’s MatchIt for PSM. All statistical tests were two-sided and used an alpha = 0.05 as the threshold for statistical significance.

### Qualitative Data

#### Setting and Participants

11 of the 38 PA MIECHV-funded programs were selected for the qualitative study, chosen to supply a representative sample of agencies, based on program size, location, and model type, including NFP, PAT, EHS, and Healthy Families America (HFA, which, due to data constraints, could only be included in the qualitative study). Program staff were interviewed during day-long site visits; enrolled clients, recruited with flyers and help from program staff, were interviewed over the phone. Participants were verbally consented before participating in interviews lasting between 30 and 60 min. Clients were sent a $20 gift card in appreciation for their time. Interviews took place between 2013 and 2015.

#### Measures and Analysis

The interdisciplinary project team worked with home visiting leadership to develop three distinct interview guides for program administrators, home visitors, and clients, each including questions on specific program outcomes (e.g. child maltreatment; See Online Appendix C for questions that elicited content related to child maltreatment.). De-identified transcripts were imported into NVivo 10 for coding and analysis. We used a modified Grounded Theory approach to coding (Glaser and Strauss 1967), including a priori codes relating to quantitative metrics included in the evaluation. Using a constant comparative approach, coders met regularly to review memos and coding comparison queries to discuss and refine node definitions and the application of codes. Discrepancies were resolved through group consensus. A thematic analysis was conducted on all interview content related to child maltreatment.

## Results

### Quantitative Data

#### Cohort Demographics

The entropy balanced NFP cohort included 8736 clients enrolled in 22 NFP programs between 2008 and 2014 matched to 165,033 comparisons. The propensity score matched PAT cohort included 851 clients enrolled in nine PAT programs matched to 2929 comparisons; EHS cohort included 866 clients enrolled in seven EHS programs propensity score matched to 3100 comparisons (Table 1).

**Table 1:** Characteristics of clients of nurse–family partnership (NFP), parents as teachers (PAT), and early head start (EHS), and comparison women, 2008–2014

	NFP				PAT				EHS			
	Comparison women		Clients		Comparison women		Clients		Comparison women		Clients	
	N = 165,033	%	N = 8736	%	N = 2929	%	N = 851	%	N = 3100	%	N = 866	%
Age, < 18 years	15,601	20.1	1955	22.4	183	6.5	51	6.0	185	5.8	54	6.2
Race/ethnicity												
White	67,240	47.8	3028	47.4	2252	77.3	657	77.9	1506	50.8	420	49.1
Black	32,837	22.3	1451	22.7	251	8.4	72	8.5	987	30.5	275	32.1
Hispanic	25,232	28.2	1801	28.2	365	12.7	107	12.7	483	15.6	139	16.2
Other	5751	1.8	112	1.8	50	1.6	7	0.8	91	3.0	22	2.6
Unmarried	131,010	89.9	7857	89.9	2206	74.8	584	68.6	2480	78.3	657	75.9
Education, less than high school	37,307	37.9	3347	38.3	825	28.3	258	30.3	1123	36.3	322	37.2
TANF receipt	54,399	43.2	3770	43.2	1546	51.9	411	48.3	1676	52.5	446	51.5
Foodstamp receipt	55,753	49.0	4280	49.0	1882	63.3	517	60.8	1899	60.2	495	57.2
Depression in proximity to pregnancy	3820	6.0	527	6.0	287	10.6	113	13.3	162	5.3	69	8.0
Substance abuse in proximity to pregnancy	3599	3.2	280	3.2	166	5.9	46	5.4	128	4.1	40	4.6
Medicaid eligibility in proximity to pregnancy	55,664	40.3	3534	40.5	982	32.8	266	31.3	924	29.8	181	20.9
Smoking, prior to pregnancy	46,168	30.7	2684	30.7	1361	46.3	382	44.9	1321	42.5	350	40.4

Table reports unweighted Ns and weighted proportions

Across all models, the majority of clients were unmarried and non-Hispanic white race/ethnicity. In terms of model-specific differences, compared to PAT and EHS client, NFP clients were most likely to be under the age of 18, most likely to be Hispanic, and least likely to smoke prior to pregnancy (Table 1).

#### Abuse Episodes Among Clients and Comparison Women

Across all models, children of clients were significantly more likely to experience an abuse episode than comparisons: NFP OR: 1.32, 95% CI [1.08, 1.62]; PAT OR: 4.11, 95% CI [1.60, 10.55]; EHS OR: 3.15, 95% CI [1.41, 7.06] (Table 2). Within NFP, 1.4% of client children (n = 120) and 1.0% of comparison children (n = 1488) sustained an abuse injury within 24 months of life; 1.1% (n = 9) of PAT-enrolled children and 0.3% of PAT comparison children (n = 9) sustained an abuse injury within 24 months of enrollment; and 1.3% of EHS program-enrolled children (n = 11) and 0.4% EHS comparison children (n = 14) sustained an abuse injury within 24 months of enrollment.

**Table 2:** Marginally standardized probabilities and odds of child abuse among comparison women and home visiting clients enrolled in nurse–family partnership (NFP), parents as teachers (PAT), and early head start (EHS)

HV program	Comparisons (%)	HV clients (%)	OR (95% CI)	p Value
NFP	1.0	1.4	1.32 (1.08, 1.62)	0.008
PAT	0.3	1.1	4.11 (1.60, 10.55)	0.003
EHS	0.4	1.3	3.15 (1.41, 7.06)	0.005

#### Distribution of Injury Types among Client Children with an Abuse Episode

The most frequent injury types among home visited children who experienced an abuse episode included superficial injuries and dislocation, fracture or crush injuries. Burns were the least prevalent injury type in aggregate. While poisonings were infrequent among NFP children with abuse episodes (2.0%), one in five children in PAT with abuse episodes experienced poisoning while dislocations, fractures and crush injuries occurred with half the frequency among PAT children than NFP children with abuse (23.5 versus 59.7%) (Table 3).

**Table 3:** Marginally standardized probabilities of injury types among children with an abuse episode by home visiting program

HV program	Marginally standardized probability (%)
NFP	
Superficial injury	38.1
Dislocation, fracture, crush	59.7
Poisoning	2.0
Burns	3.8
PAT	
Superficial injury	30.4
Dislocation, fracture, crush	23.5
Poisoning	19.4
Burns^a^	–
EHS	
Superficial injury	32.0
Dislocation, fracture, crush	65.3
Poisoning^a^	–
Burns^a^	–

*NFP* nurse family partnership, *PAT* parents as teachers, *EHS* early head start

^a^Due to small sample size, OR and marginally standardized probability cannot subsequently be calculated

#### Sensitivity Analyses

The prevalence of maternal childhood CPS involvement (prior to pregnancy) was equitable between clients and comparisons, with greater than one-third of mothers having CPS involvement prior to their first pregnancy. The results of sensitivity analyses demonstrate a lack of confounding by CPS involvement on the relationship between program enrollment and abuse (Online Appendix D).

Roughly 1% of NFP and PAT clients and comparisons were diagnosed with IPV in the pregnancy period (Online Appendix E, Table 1). In sensitivity analyses, maternal IPV exposure was not found to be a confounder of the relationship of program enrollment and abuse (Online Appendix E). However, across both programs, mothers with diagnosed IPV were significantly more likely to have a child with an abuse episode than those without diagnosed IPV (NFP: 2.7 versus 1.2%, p = 0.027; PAT: 6.0 versus 0.6%, p = 0.027) (Online Appendix E, Table 2).

### Qualitative Data

A total of 150 interviews were conducted with program administrators (N = 25), agency staff (N = 49), and home visiting clients (N = 76). The sample represents all four MIECHV-eligible evidence-based programs in PA, with greater representation from NFP (35% of staff and 47% of clients). Participants from urban and rural sites were equally represented (Table 4).

**Table 4:** Demographics of interview participants

	Clients (N = 76)	Staff (N = 74)
Program	%	%
NFP	47	35
PAT	24	22
EHS	14	24
HFA	14	20
Urbanicity		
Urban	51	59
Sex		
Female	93	99
Race		
White	57	82
Black/African American	33	9
Other	11	5
Ethnicity		
Non-Hispanic	95	95
Age		
18 and under	4	–
19–22	21	–
23+	75	–
Employment		
Unemployed	57	–
Marital status		
Single	51	–
Married/partnered	45	–
Separated or divorced	4	–
Education		
High school or less	50	–
Some college	37	–
College or higher	13	–
Total	100	100

Our thematic analysis of the interview data related to child maltreatment identified two primary domains: (1) how programs and staff react when there is a suspicion of child maltreatment; and (2) how programs and staff address and clients engage with child maltreatment prevention strategies.

#### Program and Staff Responses to Suspicion or Incidents of Child Maltreatment

In a few instances during interviews, clients and home visitors described instances of potential child maltreatment, shedding light on how the program responds in such circumstances (Table 5).

**Table 5:** Qualitative interview data representing concern for child maltreatment

Perpetrator	Program response	Excerpt from interview data
Both parents	Adapt program curriculum to specific child need	“I do have one family that we’re working mainly on gross motor because he’s not – when he runs he doesn’t bend his knees quite as often. And that’s due to they’re kind of putting him in the playpen and not letting him out because he’ll make a mess and they don’t feel like cleaning up the mess, obviously. So he’s kind of slowly developing gross motor because he’s being confined. So I’m trying to delicately word – in that maybe it’s because he’s in the playpen that his gross motor skills aren’t developing. So when I go over, we kind of do more activities towards that, and work that in with what I already have planned.” EHS Home Visitor, 402
Parent (non-client)	Provide informational support Adapt sessions to ask about relationship with Father CPS referral	“[I]f [my son] looks different for some reason or he comes back from his dad’s and I have a visit with her. And she’s like, he has another head contusion from his dad. [...] She really tells me – well, did you take him to the emergency room? Make sure you watch it. […] She gives me paperwork on the head contusion. What to look for – if he sleeps too much or – stuff like that. […] She gave me – I forgot what it’s called. Something Domestics – Children and Youth. But, I mean, it’s not like – I don’t think he’s harming my son. It’s just the fact that he isn’t very good at watching him. So – oh, yeah, I’ve thought about it and definitely think that next time that he does come home with a head contusion, then – or if he took him to the emergency room where he has a huge boonie on his head. Usually if he does have a boonie on his head, that’s a thick bruise or swollen, I take him straight to the emergency room right when I pick him up. We don’t go home. We don’t go eat. We go to the emergency room, just because I want to have that documented as soon as possible – and if anything is really like more internally wrong. But I would definitely – she’s given me stuff about Children and Youth.” NFP Client, 5007
Sibling (non-client)	Adapt program curriculum to specific child need	“[My son] was really aggressive towards [my daughter…]. [My son] was basically the only child that I was dealing with one-on-one. And then got pregnant having my daughter. And he started feeling neglected, started to act out more, so where he would start trying to hit her and do certain little things to her and stuff like that. And with the program, they basically helped me focus on him and have time with him and also bond with the baby. Also, help him bond with the baby, like play with – show him ways to play and stuff. [...] Basically, redirecting him to do something else, like to move him away from her, pull him aside and play with just him, like one-on-one with him.” PAT Client, 8004
Sibling (non-client)	Provide resource Provide informational and emotional support	“I have two stepchildren and the one, their mother has filled their head with a lot of awful things, and he’s very abusive and we have a lot of problems. [...O]ne day she came and [my son] had a bruise on his cheek. And she said, how did that happen? And I explained to her that he had been over playing by the baby gate and his brother come out and just hit him in the face with the baby gate. And [...] then she had seen that I had a bruise on my face where he had hit me. And she had said, this has got to stop, this is what we’re gonna do. And she brought in [a therapist] to talk to me. And then we went from there and started informing all these agencies. And she said it might now help you, but at least it’s out there that if something major happens, you attempted to try to get the help you needed and the mother closed it down, but you attempted. And she said it’s the same thing if [Son] goes to school and they find a bruise on him, they’re gonna come after you and it may not be you, it could have been him, but you’re, you know, you have at least explained the situation and have tried to get help.” EHS Client, 6001
Grandparent (non-client)	Provide informational and emotional support	“I went through a domestic violence case between my mom and then through the girls’ dad. […My Home visitor]actually brought paperwork, and she physically worked with me of how to do things and that, or like if she wasn’t here, I could call her for advice or give her a text message for advice. It was like a full process. It helped by steps and physical help sometimes. [...] Like she showed me how – because my [daughter], she had fractured ribs from my mom. And [my home visitor] actually helped me of ways to hold her that’ll help her ease her pain, and then – It was really nice. Without it, I probably would have went insane with all the crying. [...] I asked my mom to watch my two kids at her house while I went to the hospital because I was really, really sick. And my current boyfriend at the time, he was working. And I had like nobody else to watch my kids, and I didn’t want them to get sick and see me suffer type of thing. And then we brought her back home and she wouldn’t stop crying, and we couldn’t figure out why, then we ended up taking her to the hospital, and CYS showed up at my door at 4:00 in the morning asking if we knew what was wrong with my daughter. And I broke. I bursted into tears, because I didn’t know what’s going on.” EHS Client, 10003
Fiancé (non-client)	Unknown	“So one time, we thought [my daughter] did break her leg whenever her and [my fiancé] were wrestling. But we went to [Hospital 1]. They made us wait two hours in the waiting room, so I left. And they called CYS on me, and CYS had came. And they just wanted to see that [my daughter] wasn’t afraid of [my fiancé] because the hospital was saying that the father was abusing her. They closed us out that day knowing that, you know, nothing bad was going on. So we took her to [Hospital 2] because I wasn’t waiting there if my daughter may have had – like, a one-year old may end up having a broken leg. I’m not wasting any time, so I changed the hospital. I went up to [Hospital 2] instead. Here, she didn’t have a broken leg. [...] It was, like, a fracture in her thigh bone. [...] So, like, I had told, you know, CYS, you know, I left [Hospital 1]. I went to [Hospital 2] because they were making my daughter wait in the waiting room for two hours.” EHS Client, 4005

Of the cases describing incidents concerning for abuse and neglect, most involved caregivers other than the client as the perpetrator, including partners, child siblings, and grandparents. Program responses tended to focus on supporting the client after the abuse had occurred with parenting support, referrals, and connections to resources. Some home visitors described relying on CPS to manage child maltreatment issues.

[I]f there is any indication of violence in the home that would always be referred to [CPS], if that child was being exposed to that. A lot of times, you can just see it while you’re there. […] sometimes they don’t even try to hide it. Or they’ll […] say something that the other person has been doing or whatever. So we don’t get into that too much, other than to say a healthy environment is what our goal is here and whatever you need to do to get that. HFA/PAT Home Visitor, 801

Home visitors qualified their relationship with CPS in many ways, including their role as mandated reporters. Given the negative feelings often associated with CPS, some home visitors described being careful about how they explained their relationship with CPS to clients.

#### Approach to and Engagement with Child Maltreatment Prevention

Home visitors described focusing on content related to positive parenting, secure attachment, and stress management as the curricular drivers of child abuse prevention. Many staff referenced the program training they received, which taught them that supporting the development of a strong bond between a parent and child could reduce the chances of maltreatment.

We stress with [parents] attachment and bonding. Because if a parent does attachment and bonding, we’ve learned through all of our trainings with the HFA, they’re gonna be less likely to abuse their child […], and that is what everything circles back to. HFA Home Visitor, 103

Home visitors focused with parents on approaches and frameworks for disciplining children. The described curricula addressed strategies directed both at the children, to manage their behavior, and at the adults, to manage associated stress.

[T]he program talks about strategies with kids having temper tantrums and stuff. Not only things that you can do for the kiddo, but things as a parent. Maybe you need to step away, take a few breaths, collect yourself and then go back and address the issue. EHS Administrator, 1002

Parents valued this guidance and support, perceiving improvements in managing their reactions to frustrating child behaviors. Clients attributed personal changes to the general encouragement from home visitors to attend to their own personal needs and more specific support related to stress.

I get stressed out easy and she’s helped me learn how to deal with stressful situations, when it comes down with my daughter when she starts getting in her, “give me, wanna wanna” modes […] and I get really stressed about it. She helps me learn how to deal with the stress and teaches me how to deal with my daughter […]. […S]he told me, it’s important that you do this for yourself. Also, it’s important you do this for you daughter. I need to take a little down time. HFA Client, 1004

Some clients described how learning new discipline strategies gave them the opportunity to break a cycle of abuse.

We’ve talked about discipline, not so much of abuse. […] I think it’s effective to make sure that my child understands that I know what they want and kind of having different methods instead of going directly to a punishment. […]My parents disciplined me by beating me and punishing me for extremely long periods of time […]. […] Those things that I didn’t really know about prior to being involved in the program because those are things that weren’t taught to me as a child. PAT Client, 7020

When asked how they talked with their home visitor about protecting their child from abuse, a number of clients did not remember the subject being addressed. Many clients acknowledged that discussing child maltreatment should be part of the curriculum covered by their home visitor. Implied in the absence of direct discussions of child abuse was the described assumption that the home visitor knew there was not abuse occurring within the household.

I don’t remember talking about that. I would assume that she knows that we are not abusive at all here. But I know that she shouldn’t assume, because you don’t know what happens when someone leaves. PAT Client, 2004

From the client perspective, very few barriers to broaching the topic of abuse were described. One parent postulated that using videos to broach the subject of child maltreatment was the only way the program could address it without putting parents on the defensive.

I think that the videos and the conversations are really all they can do without having somebody be like, you’re intruding or PC or social warrior. That’s a sensitive subject for a lot of people, how you’re gonna discipline your kids or whatever. NFP Client, 5002

Some home visitors discussed difficulties encouraging families to alter behaviors. The set of norms a parent develops around how adults should interact with children from years of personal experience is difficult to contend with when misaligned with home visitor communication.

Challenges are we’re not there all the time to monitor them. We can refer them, we can’t force them to go. I mean, there’s a deep history in how people discipline their children and it’s hard to break that within a couple of months. NFP/PAT Home Visitor, 710

Unless a parent is personally invested in changing their patterns, it is difficult for the home visitor to champion change alone. When parents are open to using new strategies, true influence depends on the degree to which these strategies replace problematic practices.

I learned that there’s no wrong or right way to teach your child behaving. […] Like say if she would think I’m teaching her, like I pop her or whatever, that could be okay, to pop her, but you know, also you got to like start taking stuff away and then teaching her no, and telling her don’t do stuff. NFP Client, 7015

## Discussion

This study demonstrates quantifiably increased risk of early childhood abuse-related injury among children of MIECHV clients compared to non-program enrolled comparison children across three home visiting programs (NFP, EHS, and PAT) implemented in a large state. Among client children experiencing abuse, elevated rates of fracture, dislocation, and crush injuries were seen in comparison to client children experiencing non-abuse related injuries.

Null findings on child abuse prevention to home visiting programs are not new to the field. Others have cited surveillance bias among enrolled families as a reason for the lackluster program effect on child maltreatment (Gomby et al. 1999; Olds et al. 1995). While surveillance bias may be a contributor to increased observation of abuse-related injury among client families, the bias is likely most relevant to minor injuries where healthcare seeking behavior was optional. In the case of more serious and emergent injuries, including fractures, dislocations, and crush injuries, the likelihood of a family to seek healthcare is likely less dependent on the presence of home visitor. Other considerations for the observed increased rate of abuse-related injury among home visited families include unidentified confounding that does not account for the referral bias driving higher risk clients into home visiting services. To address this concern, this study included two sensitivity analyses to account for potential confounding by the two strongest psychosocial risk factors associated with child maltreatment: maternal childhood involvement with CPS or IPV. Despite a high prevalence of maternal childhood CPS involvement among clients, this risk factor was found to be balanced between clients and comparison women and did not confound findings. Maternal IPV in pregnancy was also not an identified confounder in this study, though diagnosed rates of IPV were low and likely represented underascertainment of true IPV risks within the cohort. Among mothers with diagnosed IPV in pregnancy, rates of abuse were higher even after adjustment for program enrollment, indicating that these families may be a high risk subgroup of home visited clientele.

A strength of this study is the provision of qualitative contextual support for the development of hypotheses of why home visiting programs may struggle in reducing rates of child abuse. The instances of child abuse assessed in interviews described events where harm occurred to the child outside of the client’s oversight while under the supervision of a non-client caregiver. Acknowledging the limitation that interviewees may be more likely to discuss incidents in which they were not the perpetrator, the number of examples including other caregivers is still notable. The data are reflective of other information described by program sites suggesting that the vast majority of serious and abuse-related injuries take place while the child is in the care of a non-client caregiver, often an intimate partner. As programs encourage clients to engage in educational and professional advancements, it is important to address resultant childcare needs. Additionally, the role of non-clients in child maltreatment events makes evident the importance of delivering program curriculum to as many of the caregivers in contact with children as possible. Further support for this hypothesis is demonstrated by the quantitative sensitivity analysis of the significant impact of the presence of IPV on child abuse.

The curriculum related to child maltreatment, as well as home visitors’ delivery of it also plays a role in achieving intended program outcomes. Recent evidence suggests that home visiting programs may struggle to achieve positive child maltreatment outcomes due to ineffectual program delivery. Much of home visiting curricula is prepared to help parents decrease the effects of adverse childhood experiences (ACEs) on their children, even without a reduction in actual ACEs (McKelvey et al. 2016). However, home visitors themselves have not reported high efficacy in guiding discussions with parents related to sensitive topics such as maltreatment and violence (Duggan et al. 2007). Casillas et al. notes that child maltreatment outcomes in particular are negatively affected by low home visitor efficacy and fidelity in service provision (Casillas et al. 2016). Our qualitative data demonstrate variations in whether and how home visitors discussed child abuse with clients, such that a number of clients did not recollect the topic ever being raised. This finding may have been driven by role issues, as mandatory reporters and assumed affiliations with CPS were barriers to direct discussions of maltreatment. Home visitors also struggled against normative factors shaping behavior change, as well as those more directly related to maltreatment such as corporal punishment. A recent evaluation of PAT program in Connecticut demonstrated significant reductions in substantiated child maltreatment; however, this finding was driven by reductions in neglect with no demonstrated impact on child abuse. Such findings further highlight the need for additional supports around abuse specifically, but also provide support for qualitative findings in this study that suggest more home visitor comfort in the domains of parenting that may be more directly related to neglect-related maltreatment (Chaiyachati et al. 2018).

Despite hearing an openness to discussing issues of child maltreatment from more clients than those noting challenges, overall the qualitative data illustrate little direct engagement around child maltreatment. Interviews with clients and staff demonstrate that indirect approaches, such as activities to alter parenting styles or decrease maternal stress, are present and more likely to be delivered with fidelity. However, it is possible that focusing on supporting positive parenting and stress reduction is not sufficient to effectively reduce the risk of abuse incidents among the subset of home visited families most at-risk for maltreatment.

Finally, the qualitative data describe limited reactive responses to situations concerning for neglect and referral to outside resources—mainly CPS. The data highlights the dependence of home visiting success on the system of services in which the program resides. The importance of the quality, access, and connectedness of CPS, childcare, and healthcare to home visiting services towards the prevention of child maltreatment prevention cannot be overstated. The ability of home visitors to transition from reactivity to proactivity around child abuse is dependent on effective referral relationships, trust in the CPS system, and timely and affordable access to services that mitigate abuse risks, including childcare and healthcare.

This study has several limitations related to study design and data availability and reliability. The observational study design is subject to bias when estimating program effects; however the use of propensity score and entropy matching, which mimic randomization and control for measured differences between clients and comparisons and sensitivity analyses with likely confounders, minimizes this concern and is a widely accepted technique for estimating causality when randomization is not feasible or appropriate. Moreover, the standardized approach to outcome ascertainment using administrative rather than self-reported data coupled with the rigorous quasi-experimental design facilitate standardized interpretation of findings across three home visiting models and numerous diverse implementing sites. While our analyses were unable to take program dosage and the perpetrator of abuse incidents into account in a standardized fashion as this information was unavailable in administrative data files, this information was gleaned from qualitative interviews when it was addressed by home visitors or clients. The study is not able to account for surveillance bias; however, the study used an intention to treat design to observe children for two full years following enrollment while a significant proportion of families are not retained in services for this duration (Burwick et al. 2014). Lastly, given the sensitive nature of this topic, the qualitative interview data is likely impacted by social desirability bias.

## Conclusion

Home visiting programs have a stated objective of preventing child abuse-related injury among high-risk families. The success of abuse prevention depends on the strength of the curriculum, the fidelity of delivery, and whether it reaches the people in caregiving roles. Given that home visitors depend greatly on other community agencies and public systems, it is naïve to expect home visiting to achieve strong outcomes without a well-integrated and -resourced service network. Programs and clients would benefit from curriculum that more directly and proactively addresses child maltreatment, as well as knowledge of and access to quality childcare options.

## Electronic supplementary material

Below is the link to the electronic supplementary material.

Supplementary material 1 (DOCX 26 KB)
