# Correction to: A Mixed Methods Evaluation of Early Childhood Abuse Prevention Within Evidence-Based Home Visiting Programs

**DOI:** 10.1007/s10995-018-2623-x

**Published:** 2018-08-28

**Authors:** M. Matone, K. Kellom, H. Griffis, W. Quarshie, J. Faerber, P. Gierlach, J. Whittaker, D. M. Rubin, P. F. Cronholm

**Affiliations:** 1 PolicyLab, The Children’s Hospital of Philadelphia, Philadelphia, PA USA; 2 Division of General Pediatrics, The Children’s Hospital of Philadelphia, Philadelphia, PA USA; 3 Perelman School of Medicine, University of Pennsylvania, Philadelphia, PA USA; 4 Mixed Methods Research Lab, University of Pennsylvania, Philadelphia, PA USA; 5 Department of Family Medicine and Community Health, University of Pennsylvania, Philadelphia, PA USA; 6 Center for Public Health Initiatives, University of Pennsylvania, Philadelphia, PA USA; 7 Leonard Davis Institute of Health Economics, University of Pennsylvania, Philadelphia, PA USA; 8 Roberts Center for Pediatric Research, 2716 South Street, Philadelphia, PA 19146 USA

## Correction to: Maternal and Child Health Journal 10.1007/s10995-018-2530-1

The article “A Mixed Methods Evaluation of Early Childhood Abuse Prevention Within Evidence-Based Home Visiting Programs”, written by M. Matone, K. Kellom, H. Griffis, W. Quarshie, J. Faerber, P. Gierlach, J. Whittaker, D. M. Rubin and P. F. Cronholm, was originally published electronically on the publisher’s internet portal (currently SpringerLink) on 31 May 2018 without open access. With the author(s)’ decision to opt for Open Choice the copyright of the article changed on 27 July 2018 to © The Author(s) 2018 and the article is forthwith distributed under the terms of the Creative Commons Attribution 4.0 International License (http://creativecommons.org/licenses/by/4.0/), which permits use, duplication, adaptation, distribution and reproduction in any medium or format, as long as you give appropriate credit to the original author(s) and the source, provide a link to the Creative Commons license and indicate if changes were made.

The original article has been corrected.

