# Supplementary material for: A Mixed Methods Evaluation of Early Childhood Abuse Prevention Within Evidence-Based Home Visiting Programs
Source: Matern Child Health J. 2018 May 31;22(Suppl 1):79–91. doi: 10.1007/s10995-018-2530-1 (PMC6153766; doi:10.1007/s10995-018-2530-1)
Supplement: Supplementary file 1 — Supplementary material 1 (DOCX 26 KB) [file 10995_2018_2530_MOESM1_ESM.docx]

Table of Contents

Appendix A: Methodology for abuse and injury episode creation

Appendix B: Qualifying ICD-9 codes for High-Risk Injuries (HRI)

Appendix C: Questions from the interview guides for clients, home visitors, and program administrators that elicited content related to child maltreatment

Appendix D: Distribution of comparison women and home visiting clients enrolled in Nurse Family Partnership (NFP) living in Philadelphia county with maternal involvement with child protective services (CPS) prior to childbirth

Appendix E: Marginally standardized probabilities and odds of child abuse outcome by maternal intimate partner violence (IPV) status, adjusting for maternal home visiting enrollment.

*Appendix A*

Appendix A: Methodology for abuse and injury episode creation

First, all claims for medical encounters for injury or abuse for the same child were sorted chronologically and any claims occurring within 1 day of each other were collapsed, retaining all unique diagnoses. Second, these collapsed claims were assigned Barell matrix categories (Barell, Aharonson-Daniel, Fingerhut, & MacKenzie, 2002) (Table A). Starting chronologically with the first injury-related claim (index claim), all subsequent claims for medical encounters for the same child with the same Barell matrix categorization within a 180-day interval of the index claim were assigned to a single episode (Matone et al., 2012; J.N. Wood, French, Song, & Feudtner, 2015). Third, if claim within the 180 day window did not have diagnoses mapped to any Barell matrix categorizations of the index claim, a new episode was created; that claim became a new index claim and re-started the 180-day window for collapsing claims with the same Barell category. For each episode, the index claim indicated the start date of the episode. All claims collapsed into a single episode retained all diagnosis flags. This was done to ensure that injury events requiring multiple follow-up medical visits were correctly counted as a single episode. This resulted in episodes that could have more than one type of injury if a set of related claims included more than one Barell injury categorization.

Given that particular categories may have shorter follow up times, further refinement of the 180 day interval was warranted. For episodes with claims related to superficial injury and poisoning encounters, claims occurring within 30 days were collapsed into a single episode. Similarly, claims related to dislocations/fractures within 42 days were collapsed into one episode. These windows were determined via a manual review of claims and episodes by members of the study team.

## Appendix B

| Appendix B  Qualifying ICD-9 codes for High-Risk Injuries (HRI) | |  |  |  |
| --- | --- | --- | --- | --- |
| Category | ICD-9 Code |  |  |  |
| Femur Injury | 820.00, 820.01, 820.02, 820.03, 820.09, 820.10, 820.11, 820.12, 820.13, 820.19, 820.20, 820.21, 820.22, 820.30, 820.31, 820.32, 820.3x, 820.8x, 820.9x, 821.00, 821.01, 821.10, 821.11, 821.20, 821.21, 821.22, 821.23, 821.29, 821.30, 821.31, 821.32, 821.33, 821.39 |  |  |  |
| Rib Fracture | 807.0-807.19 |  |  |  |
| Radius/Ulna Fracture | 813.0-813.93 |  |  |  |
| Tibia/Fibula Fracture | 823.0-823.92 |  |  |  |
| Humerus Fracture | 812.0-812.59 |  |  |  |
| Traumatic Brain Injury (TBI) | 800.1x, 800.2x, 800.3x, 800.4x, 800.6x, 800.7x, 800.8x, 800.9x, 801.1x, 801.2x, 801.3x, 801.4x, 801.6x, 801.7x, 801.8x, 801.9x, 803.1x, 803.2x, 803.3x, 803.4x, 803.6x, 803.7x, 803.8x, 803.9x, 804.1x, 804.2x, 804.3x, 804.4x, 804.6x, 804.7x, 804.8x, 804.9x, 851.xx, 852.0x, 852.1x, 852.2x, 852.3x, 852.4x, 852.5x, 853.0x, 853.1x |  |  |  |

## Appendix C

Appendix C

| *Questions from the interview guides for clients, home visitors, and program administrators that elicited content related to child maltreatment* | |
| --- | --- |
| We asked clients: | |
| Tell me about what you and your home visitor talked about related to child safety and accidents and injury. | |
| Probe: | What did you take away from that part of the program? |
|  | How much has this been an issue for you now or in the past? |
|  | How, if at all, has that information changed how you do things? |
|  | How important is it for programs like these to focus on this issue? |
|  | How effective do you think the program is at helping people with this issue? |
|  | What gets in the way of helping people with this issue? |
| Tell me about what you and your home visitor talked about related to protecting your child from abuse. | |
| Probe: | What did you take away from that part of the program? |
|  | How much has this been an issue for you now or in the past? |
|  | How, if at all, has that information changed how you do things? |
|  | How important is it for programs like these to focus on this issue? |
|  | How effective do you think the program is at helping people with this issue? |
|  | What gets in the way of helping people with this issue? |
| Tell me about what you and your home visitor talked about related to conflict or unhealthy relationships between parents or family members. | |
| Probe: | What did you take away from that part of the program? |
|  | How much has this been an issue for you now or in the past? |
|  | How, if at all, has that information changed how you do things? |
|  | How important is it for programs like these to focus on this issue? |
|  | How effective do you think the program is at helping people with this issue? |
|  | What gets in the way of helping people with this issue? |
| We asked staff: | |
| Can you describe the population you work with? | |
| Probe: | What are the biggest needs of the families you serve? What needs do your clients have that your community cannot provide? |
| Now tell us about how you get to know your families and set up visits. | |
| Probe: | How do you decide what you are going to work on with your families? Describe your clients’ needs when they first start the program. How do clients’ needs tend to change over time? |
|  | Describe the core parts to the curriculum you follow (i.e. what makes your curriculum PAT / NFP / HFA / EHS). |
|  | What ability do you have to change the curriculum if necessary to meet your client’s specific needs? What is usually going on that triggers the need to make changes in the curriculum? How often do home visitors change the way the either assess the needs of clients of use the curriculum to meet the needs of the client? |
| Early childhood programs are often evaluated based on their ability to affect factors shaping children’s health such as smoking cessation, avoiding low birth weight babies, using the emergency department, and preventing child abuse/injury: | |
| Probe: | How much of an issue are these outcomes for the clients you serve?  How do you perceive your program’s ability to affect these outcomes? Do you feel more effective with some of these outcomes compared to others? |
|  | What parts of your curriculum address these issues? |
|  | What are the challenges to addressing these issues with the clients you serve? |
| Thinking about the work that you do with families, how do the successes you think are important compare to the outcomes we just discussed? | |
| Tell me about the training you have received related to your job. | |
| Probe: | What occurs, where, and how often? |
|  | How prepared did you feel for the job when you started? How has your comfort changed over time? |

*Appendix D*

| Appendix D  Table 1  *Distribution of maternal involvement with the child protective services (CPS) prior to childbirth among comparison women and home visiting clients enrolled in Nurse Family Partnership (NFP) living in Philadelphia county.* | | | | | | | | |
| --- | --- | --- | --- | --- | --- | --- | --- | --- |
|  | Comparison Women | | | | Clients | | | |
|  | N=3,936 | | % | | N=1,035 | | % | |
| Maternal CPS History |  | |  | |  | |  | |
| No | 2,532 | | 64.3 | | 660 | | 63.8 | |
| Yes | 1,404 | | 35.7 | | 375 | | 36.2 | |
|  |  | |  | |  | |  | |
|  |  | |  | |  | |  | |
|  | | | | | | | | |
| Table 2  *Unadjusted and adjusted^a^ odds of child abuse outcome among comparison women and home visiting clients enrolled in Nurse Family Partnership (NFP) living in Philadelphia county.* | | | | | | | | |
| Injury Type | | Unadjusted OR (95% CI) | | p-value | | Adjusted OR (95% CI) | | p-value |
| Abuse | | 0.94 (0.52, 1.69) | | 0.830 | | 0.93 (0.52, 1.67) | | 0.804 |
| *^a^ Adjusted for maternal involvement with child protective services prior to childbirth (y/n).* | | | | | | | | |

*Appendix E*

| Appendix E  Table 1  *Distribution of maternal intimate partner violence diagnosed in the pregnancy period among home visiting clients and comparison women.* | | | | |
| --- | --- | --- | --- | --- |
|  | Comparison Women | | Clients | |
| Maternal IPV | N | %^b^ | N | %^b^ |
|  |  |  |  |  |
| NFP*^a^* |  |  |  |  |
| No | 163,891 | 99.3 | 8,640 | 98.6 |
| Yes | 1,142 | 0.07 | 96 | 1.1 |
| PAT*^a^* |  |  |  |  |
| No | 2,904 | 99.1 | 843 | 99.1 |
| Yes | 25 | 0.9 | 8 | 0.9 |
| *^a^NFP (Nurse Family Partnership); PAT (Parents as Teachers)*  *^b^Weighted percentages from propensity score (PAT) and entropy balanced (NFP) cohorts.*  *The time period from date of conception through the first month of the child’s life was used to observe presence of IPV.*  *Outcome not estimable for EHS because there are zero instances of maternal IPV preceding a child receiving an abuse diagnosis.* | | | | |

| Table 2  *Marginally standardized probabilities and odds of child abuse outcome by maternal intimate partner violence (IPV) status, adjusting for maternal home visiting enrollment.* | | | | |
| --- | --- | --- | --- | --- |
| HV Program | No IPV (%) | IPV (%) | OR (95% CI) | p-value |
| NFP | 1.2 | 2.7 | 2.31 (1.09, 4.85) | 0.027 |
| PAT | 0.6 | 6.0 | 10.61 (1.32, 85.55) | 0.027 |
|  |  |  |  |  |
| *The time period from date of conception through the first month of the child’s life was used to observe presence of IPV.*  *Outcome not estimable for EHS because there are zero instances of maternal IPV preceding a child receiving an abuse diagnosis.* | | | | |
